# NADH Intraperitoneal Injection Prevents Lung Inflammation in a BALB/C Mice Model of Cigarette Smoke-Induced Chronic Obstructive Pulmonary Disease

**DOI:** 10.3390/cells13100881

**Published:** 2024-05-20

**Authors:** Nada Slama, Amina Abdellatif, Karima Bahria, Sara Gasmi, Maamar Khames, Abderrahmene Hadji, George Birkmayer, Mustapha Oumouna, Yassine Amrani, Karine Benachour

**Affiliations:** 1 Laboratory of Experimental Biology and Pharmacology, Faculty of Sciences, Dr. Yahia Fares University, Medea 26000, Algeria; nadaslama94@gmail.com (N.S.); amina.abdellatif.bio@gmail.com (A.A.); karima_bhr@hotmail.com (K.B.); sarah_gasmi@yahoo.fr (S.G.); drmammar@hotmail.fr (M.K.); abdouchiwa@hotmail.fr (A.H.); oumouna@gmail.com (M.O.); 2 Department of Medical Chemistry, University of Graz, 8020 Graz, Austria; 3 Birkmayer Laboratories, 1090 Vienna, Austria; 4 Department of Respiratory Sciences, Institute of Lung Health and NIHR Leicester BRC-Respiratory, Glenfield Hospital, University of Leicester, Leicester LE1 7RH, UK; ya26@leicester.ac.uk

**Keywords:** cigarette smoke, animal model, NADH, oxidative stress, inflammation

## Abstract

Cigarette smoke is one of the main factors in Chronic Obstructive Pulmonary Disease (COPD), a respiratory syndrome marked by persistent respiratory symptoms and increasing airway obstruction. Perturbed NAD+/NADH levels may play a role in various diseases, including lung disorders like COPD. In our study, we investigated the preventive effect of NADH supplementation in an experimental model of COPD induced by cigarette smoke extract (CSE). N = 64 mice randomly distributed in eight groups were injected with NADH (two doses of 100 mg/kg or 200 mg/kg) or dexamethasone (2 mg/kg) before being exposed to CSE for up to 9 weeks. Additionally, NADH supplementation preserved lung antioxidant defenses by preventing the functional loss of key enzymes such as superoxide dismutase (SOD), glutathione peroxidase (GPX), catalase, and the expression levels of glutathione (GSH) (*n* = 4, *p* < 0.001). It also reduced oxidative damage markers, such as malondialdehyde (MDA) and nitrites (*n* = 4, *p* < 0.001). A marked increase in tissue myeloperoxidase activity was assessed (MPO), confirming neutrophils implication in the inflammatory process. The latter was significantly ameliorated in the NADH-treated groups (*p* < 0.001). Finally, NADH prevented the CSE-induced secretion of cytokines such as Tumor Necrosis Factor alpha (TNF-α), IL-17, and IFN-y (*n* = 4, *p* < 0.001). Our study shows, for the first time, the clinical potential of NADH supplementation in preventing key features of COPD via its unique anti-inflammatory and antioxidant properties.

## 1. Introduction

Chronic obstructive pulmonary disease (COPD), a condition that affects 328 million people worldwide, is one of the most frequent and life-threatening non-communicable, preventable, and irreversible chronic lung diseases. The Global Burden of Disease report states that COPD is the third leading cause of death in the world [1,2]. COPD is characterized by chronic inflammation that causes damages to the airways, alveoli, and pulmonary vessels, ultimately leading to airflow limitation [3]. COPD is mainly the result of harmful environmental stimuli, including cigarette smoke, which leads to excessive inflammatory responses in the lung, abnormal tissue repair, and the destruction of lung tissue [4]. Cigarette smoking has a well-known negative health impact; it comprises around 4500 components in its gaseous and particle phases. These substances include direct carcinogens, poisons, and oxidants [5].

The mechanisms by which cigarette smoke plays a role in COPD pathogenesis are likely to be complex, but accumulating evidence suggests a potential role of mitochondrial dysfunction [6]. Mitochondria has significant functions in the lung during homeostasis. Various key functions in epithelial cells, such as surfactant synthesis, cellular senescence, mucociliary activity, and mucus secretion, can all be controlled by mitochondria [7]. Mitochondrial structural and functional perturbations have been involved in the pathogenesis of various lung diseases, including COPD. These disturbances alter cell energy as well as different essential cellular homeostatic functions in which mitochondria are known to be implicated, such as mucus secretion, surfactant production regulation, and immunologic defenses [7,8]. Nicotinamide adenine dinucleotide (NAD) is a ubiquitous intracellular electron transporter that has been involved in the majority of aging and neurodegenerative processes. NAD may be present in cells in both its oxidized (NAD+) and reduced forms (NADH) [9]. Their ratio controls the intracellular redox environment, antioxidant capability, and cell signaling and it is known to upregulate anti-inflammatory molecules in various chronic diseases [10]. In this context, an altered mitochondrial metabolism can reduce NADH production, raising the NAD+/NADH ratio, and impairing antioxidant activity, leading to increased oxidative stress and cell dysfunction [11]. Indeed, it has been reported that the intracellular NAD+/NADH level was decreased in various chronic diseases associated with an increased oxidant burden, such as diabetes [12], neurodegenerative diseases [13], and idiopathic lung fibrosis [14]. This downregulation has been linked to the hyperexpression of the NAD+-consuming poly-(ADP-ribose) polymerase 1 (PARP-1), an enzyme with numerous cellular functions, such as cell death and DNA repair function. However, in pathological states associated with intense DNA damage, PARP-1 has been described as one of the main mechanisms driving the cellular loss of NAD+ and ATP, resulting in cell death [13]. Manipulating NADH levels using pharmacological agents has the potential to restore mitochondrial respiration and has shown promising results in the treatment of diabetes and multiple disorders [12,15], chronic fatigue syndrome [16], and neurodegenerative diseases such as Parkinson’s disease [17] and Alzheimer’s [18], but this has not yet been studied in the context of COPD.

Accordingly, the present work was designed to evaluate the effects of NADH treatment in an experimental model of cigarette smoke extract (CSE)-induced COPD in *BALB/C* mice.

## 2. Materials and Methods

Animals: Female *BALB/C* mice weighing 20–23 g were obtained from the Institute Pasteur of Algiers, Algeria. Before exposure, mice spent a week getting used to the environment. Throughout the duration of the trial, mice were given free access to water and conventional rodent food (ONAB, El-Kseur, Medea district, Algeria). This study was performed according to the guidelines established by the Institutional Animal Care and Use Committee (IACUC) at the University of Medea (no. IACUC-CEEA-02).

Reagents: NADH was provided by Birkmayer NADH GmbH (Vienna, Austria); Ellman’s reagent (DTNB), Pyrogallol, and other solvents and chemicals were bought from Sigma Chemicals (St. Louis, MO, USA). Acetic acid, ethanol, and other reagents were purchased from Biochem Chemopharma (Cosne-Cours-sur-Loire, France).

Preparation of Cigarette Smoke Extract (CSE): CSE was prepared by a modification of the technique described in previous studies [19]. Briefly, commercial cigarettes (Nassim) were used in this study. Three cigarettes (0.8 mg nicotine, 12 mg tar) were burned and bubbled into 3 mL of PBS. The mainstream of smoke, including tars and nicotine, was almost completely removed using a force-feeding syringe (60 mL). The CSE extracts were prepared fresh prior to each experiment and used for administration after adjusting the pH to between 7.00 and 7.40 and filtering through a 0.2 μm pore filter. CSE preparation was standardized by measuring the absorbance at a wavelength of 320 nm.

Experimental design: The animals were randomly divided into eight groups, each with its own set of conditions: the control group, CSE group, Dex (2 mg/kg/2 days) group + CSE, NADH (100 mg/kg/11 days) + CSE group, NADH (100 mg/kg/5 days) group, NADH (200 mg/kg/5 days) + CSE group, NADH (100 mg/kg/5 days) alone group, and Dex (2 mg/kg/2 days) alone group. NADH was administered by intraperitoneal injection every 11 days (11-day regimen) or 5 days (5-day regimen). All mice except those in the control group were intraperitoneally injected with NADH/dexamethasone (0.2 mL/kg) 30 min before CSE/saline (0.3 mL). Body weights were noted every 11 days, and all mice were sacrificed on day 60 after the initial CSE treatment.

Complete blood count in whole blood: EDTA-coated microcentrifuge tubes were used to collect blood from the tail at the end of each experiment. An initial sample volume of 50 μL of whole blood was required for analysis using RaytoRT7600S (Shenzhen, China), hematology analyzer which counts total white blood cells, lymphocytes, granulocytes (neutrophils, eosinophils, or basophils), and MID (monocytes and all non-granulocytes or lymphocytes blood cells).

Assessment of oxidative stress and antioxidant defense in lung homogenates: Isolated lung lobes were homogenized in 1 mL of cold PBS using a homogenizer (IKA ultraturrax, IKA, Wilmington, NC, USA) and centrifuged for 15 min at 6000 rpm to collect tissue supernatants. Protein concentration in the supernatants was calculated according to the Bradford method [20]. To determine the antioxidant effect of NADH in our COPD animal model, we measured antioxidant enzymes and oxidative biomarkers in lung homogenates using a spectrophotometer (SP-300nano) as described below.

Determination of oxidative biomarkers: Malondialdehyde (MDA) peroxidation is one of the most valid indicators to estimate lipid peroxidation. Levels of lung MDA were determined based on the method described by Ohkhawa et al. (1979), which is based on the reaction of 2-thiobarbituric acid (TBA) with MDA to form a pink chromogen, which can be measured at 532 nm [21]. MDA peroxidation was expressed as μmol/mg of total lung protein. Nitrite levels (expressed as μM) were measured in lung homogenates using the modified Griess method [22]. Myeloperoxidase (MPO) activity was measured to estimate neutrophil accumulation in lung tissue following the protocol used by Ruyssers et al., 2009 [23] and expressed as units per gram of tissue, with 1 unit equaling the amount of MPO necessary to convert 1 μmol of H_2_O_2_ to H_2_O per minute.

Determination of the activities of antioxidant enzymes: Superoxide dismutase (SOD) activity was measured according to the Marklund and Marklund method [24] based on the enzyme’s ability to inhibit pyrogallolauto-oxidation by the superoxide radical. The samples were read at 570 nm using a spectrophotometer reader. SOD was expressed as μmol/mg of total lung protein. Glutathione peroxidase (GPx) activity was measured using Flohe and Gunzler’s method [25] and expressed as μmol/mg of protein. Glutathione (GSH) levels were measured by spectrophotometry according to Weckbecker and Cory’s method [26] based on its ability to react with 5,5’-dithio-bis(2-nitrobenzoic acid) (DTNB), and expressed as μmol/mg of total lung protein [26]. Finally, catalase activity was determined according to the method by Aebi et al. and expressed as mmol/mg of total lung protein [27].

Measurement of inflammatory biomarkers in lung tissues: Lung tissues were homogenized in PBS on ice using a homogenizer (IKA ultra turrax) and centrifuged for 15 min at 6000 rpm to collect tissues’ supernatants. The supernatants were used to determine the levels of TNF-α, IFN-y, and IL-17 using a DuoSet ELISA Development kit (R&D system, Minneapolis, MN, USA) following the manufacturer’s instructions.

Histopathological examination: The lung tissues were fixed by intratracheal instillation of 10% formalin, and then dehydrated, paraffin-embedded, and cut into 4 μm sections. Hematoxylin and eosin (H&E) staining was used for general histological examination, and trichrome coloration was used to detect the presence of collagen and fibrosis. The slides were examined under an optical light microscope (Carl ZEISS Primo Star, Oberkochen, Germany). The mean linear intercept was used to calculate alveolar enlargement as described previously [28]. The lines were briefly drawn on each lung segment, the intercepts that crossed them were counted, and the mean linear intercept was determined. Image Jv1.54j was utilized to quantify both collagen and bronchial wall thickness [29].

Statistical analysis: All data were expressed as mean ± SD. Statistics were performed using Prism 8.4.2 (GraphPad Software, San Diego, CA, USA) and one-way ANOVA with Bonferroni’s correction for multiple comparisons. Differences were considered significant when *p* < 0.05.

## 3. Results

The results of the white blood cell count, oxidative and anti-oxidative markers, and histological evaluations all indicate that NADH treatment led to significant decreases in systemic inflammation and oxidative stress and provided protection against pulmonary damage and lung fibrosis.

### 3.1. Effect of NADH Treatment on Body Weights of Mice

**Figure 1:**
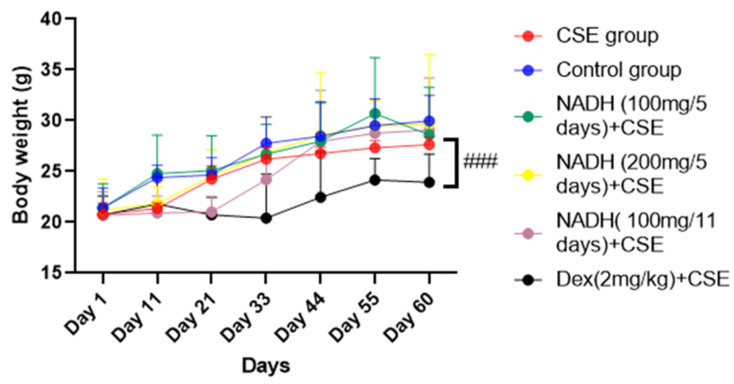
NADH effects on mice body weights. Mice were weighed every 11 days; ### *p* < 0.001 compared with CSE group (*n* = 8/per group).

As demonstrated in Figure 1, there was no significant difference in the mice’s body weights between the control group, CSE group, and NADH-treated group (*p* > 0.05). In contrast, the mice in the dexamethasone + CSE group had weights that were significantly less than those in the respective CSE group (*p* = 0.0006).

### 3.2. Effect of NADH on CSE-Induced Changes in Total White Blood Cell Count

**Figure 2:**
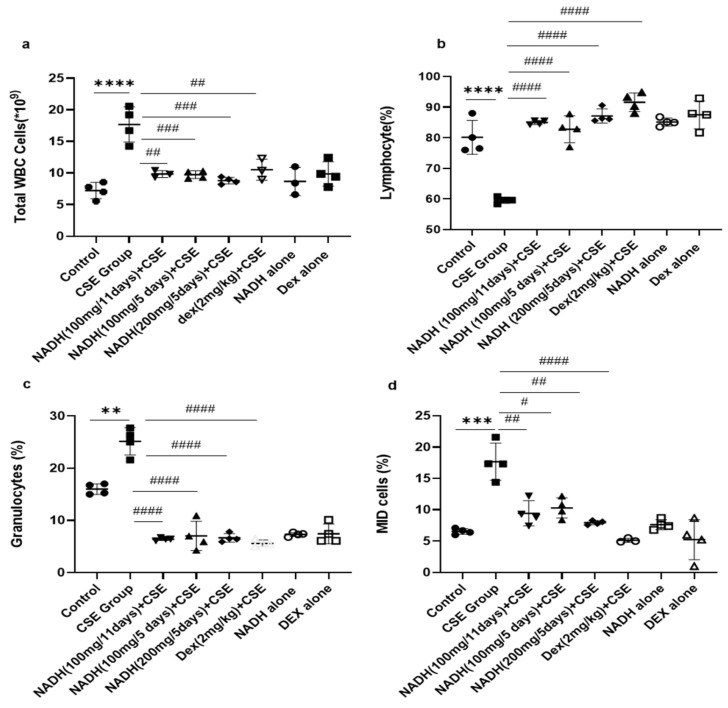
NADH, irrespective to the dose and treatment regimen, prevented blood inflammatory changes in mice exposed to CSE. Blood was collected at the end of the experiment from the control groups (diluent, dexamethasone alone, and NADH alone) and the groups treated with CSE alone or with NADH in a5-day or 11-day regimen. The numbers of total white blood cells (**a**), lymphocytes (**b**), granulocytes (neutrophils, eosinophils, or basophils) (**c**), and MID (monocyte and all non-granulocyte or lymphocyte blood cells) (**d**) were then determined as described in the Section 2. ** *p* < 0.05, *** *p* < 0.001, and **** *p* < 0.0001 compared with the control group; # *p* < 0.05, ## *p* < 0.01, ### *p* < 0.001, and #### *p* < 0.0001 compared with CSE group (*n* = 4/per group).

As revealed in Figure 2, CSE injection significantly increased the total WBC count by 2.55-fold compared with the control group (*p* < 0.0001). Irrespective of the dose (100 and 200 mg) and length of treatment (5- and 11-day regimen), NADH treatment was able to prevent all blood changes induced by CSE. NADH significantly reduced the CSE-induced increase in total WBC with a reduction average of 45.35% (Figure 2a, *n* = 4, *p* < 0.001). Dexamethasone (Dex), used as a positive control intervention for its anti-inflammatory action, resulted in a significant 41% reduction in the blood WBC count when compared to the CSE group (*n* = 4, *p* < 0.001).

The CSE group also exhibited a significant reduction (1.35-fold) in the number of lymphocytes in the blood compared to the control group (Figure 2b, *n* = 4, *p* < 0.0001). NADH and dexamethasone treatment completely prevented a CSE-induced drop in the lymphocyte number in the blood (*n* = 4, *p* < 0.0001).

CSE was also able to stimulate a significant increase in the number of total granulocytes that included neutrophils, eosinophils, and basophils from 7.22 ± 1.28 to 25.2 ± 2.7 (Figure 2c, *n* = 4, *p* = 0.0002). This significant increase was abrogated by NADH treatment with no efficacy difference between the groups (*n* = 4, *p* < 0.0001). Dexamethasone treatment also significantly decreased the granulocyte count by 77.5% when compared with the CSE group (Figure 2c, *p* < 0.0001).

CSE significantly increased blood MID cells (which reflect the white blood cells not classified as lymphocytes or granulocytes) by 2.17-fold compared to those in the control group (Figure 2d, *n* = 4, *p* = 0.008). In contrast, in all treated groups, NADH treatment led to a decrease in MID cells induced by CSE exposure (*n* = 4, 54.7% decrease). The same results were observed among the dexamethasone-treated groups, which significantly prevented a CSE-induced increase in the MID cell count by 70% when compared to the CSE group; *p* < 0.0001 (Figure 2d, *n* = 4).

We also reported that groups treated with NADH or dexamethasone alone did not exhibit any changes in the number of inflammatory cells when compared to the control group (*p* > 0.05, Figure 2).

### 3.3. Effect of NADH on CSE-Induced Lung Changes in Oxidative Stress and Antioxidant Defense Biomarkers

**Figure 3:**
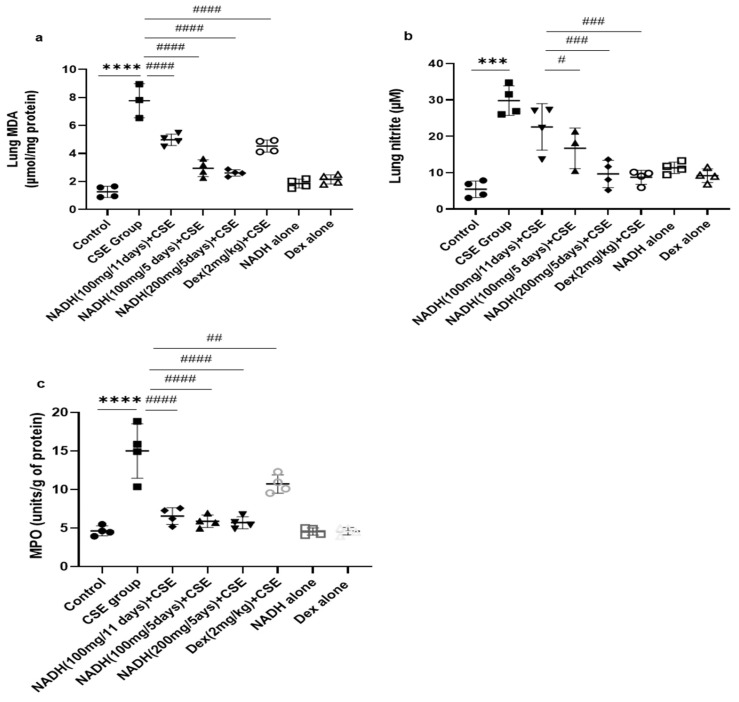
NADH reduced markers of oxidative stress in the lungs of mice exposed to CSE. The levels of malondialdehyde (MDA) (**a**), nitrites (**b**), and MPO (**c**) were assessed as described in the Section 2 in lung homogenates from the control groups (diluent, dexamethasone alone, and NADH alone) or the groups treated with CSE alone or with NADH every 5 or 11 days. *** *p* < 0.001 and **** *p* < 0.0001 versus the control group; # *p* < 0.05, ## *p* < 0.01, ### *p* < 0.001, and #### *p* < 0.0001 compared with the CSE group (*n* = 4 animals/condition).

We found that the CSE-induced COPD model was associated with a significant increase in MDA levels (6.14-fold) when compared to the control group (*p* < 0.0005, Figure 3a).

Treatment with 100 mg of NADH led to a marked reduction in the MDA levels in both treatment regimens (i.e., given every 11 and 5 days), although the effect was more pronounced when NADH was given every 5 days with 35 and 65% inhibition, respectively (*p* < 0.0001, *n* = 4/condition) or at a higher concentration of 200 mg given on a 5-day regime (*p* < 0.0001, *n* = 4/condition). The decrease in CSE-induced lung MDA levels was also inhibited by 41% by dexamethasone (*p* = 0.0188, Figure 3a).

The CSE model also led to a significant 4.7-fold increase in lung nitrite levels when compared to the control group, (*p* < 0.0001, Figure 3b). Interestingly, only the mice treated with NADH at a 100 or 200 mg dose in the 5-day regimen led to a significant reduction in nitrite levels, leading to 44% (*p* = 0.006) and 70% (*p* < 0.0001) inhibition, respectively. In contrast, 100 mg of NADH did not affect CSE-induced nitrite production in the 11-day regimen (*p* = 0.0749).

As shown in Figure 3c, CSE increased MPO activity by 3.23-fold when compared with the control group (*n* = 4, *p* < 0.0001). Interestingly, NADH treatment, irrespective of the regimen and the dose, prevented this increase by 50 to 60% (*p* < 0.0001, *n* = 4/condition). We also found that dexamethasone inhibited the CSE-induced increased MPO activity by 29% (*p* = 0.009).

**Figure 4:**
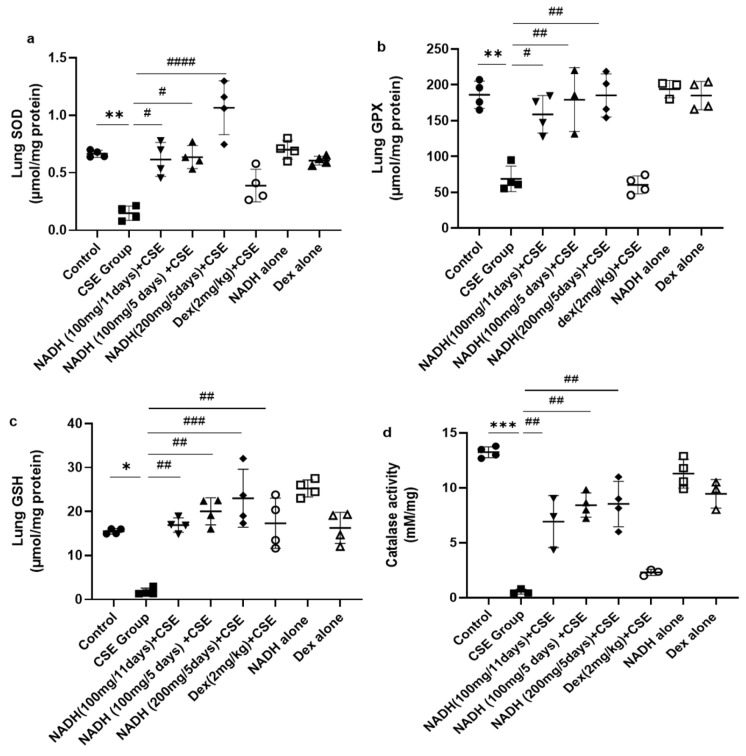
NADH prevented the reduced antioxidant response in the lungs of mice exposed to CSE. The activities of key detoxifying enzymes, including superoxide dismutase (SOD) (**a**), glutathione peroxidase (GPX) (**b**), glutathione (GSH) (**c**), and catalase (**d**), were assessed in lung homogenates from the control groups (diluent, dexamethasone alone, and NADH alone) or the groups treated with CSE alone or with NADH in the 5-day or 11-day regimen as described in the Section 2. * *p* < 0.05, ** *p* < 0.01 and *** *p* < 0.001 compared with the control group; # *p* < 0.05, ## *p* < 0.01, ### *p* < 0.001 and #### *p* < 0.0001 compared with the CSE group (*n* = 4/per group).

We also looked at the impact of CSE on the antioxidant pathways in the lungs. As demonstrated in Figure 4a, CSE caused a marked reduction (77.73%) in SOD activity in the lung (*p* = 0.0052). Interestingly, NADH, irrespective of the dose (100 and 200 mg) or treatment plan (5-day or 11-day regimen), completely prevented the loss in SOD antioxidant activity induced by CSE (*p* < 0.0001). In contrast, dexamethasone treatment failed to prevent the decrease in lung SOD activity induced by CSE (*p* = 0.1080).

NADH also exerted a similar protective effect on glutathione peroxidase (GPX), whereby the 62.98% (*p* = 0.0026) decrease in lung GPX activity induced by CSE could be completely prevented by NADH on a 5-day regimen at a 100 mg (*p* = 0.0015) or 200 mg dose (*p* = 0.0014) or 11-day regimen at 100 mg (*p* = 0.0124). In contrast, dexamethasone treatment failed to prevent the decrease in lung GPX activity induced by CSE (*p* = 0.9995) (Figure 4b).

We also assessed the impact of NADH on the levels of glutathione (GSH), one of the essential antioxidant defense pathways. The 8-fold drop in GSH levels induced by CSE (*p* = 0.0186) could be completely prevented by NADH on a 5-day regimen at 100 mg (*p* = 0.0013) and 200 mg (*p* = 0.0003) doses or on an 11-day regimen at 100 mg (*p* = 0.004). In addition, dexamethasone treatment also prevented the decrease in lung GSH activity induced by CSE (*p* < 0.05) (Figure 4c). Interestingly, NADH treatment alone appeared to augment the lung level of GSH when compared to the levels seen in the control group (*p* < 0.05) (Figure 4c).

Finally, we looked at the changes in lung catalase activity following CSE exposure and the effect of NADH. Catalase has been described as a vital antioxidant mechanism in the lungs via its ability to convert hydrogen peroxide to oxygen and water [30]. As depicted in Figure 4d, CSE reduced lung catalase activity by 95.84% (*p* < 0.0001). All NADH-treated groups, irrespective of the treatment regimen (5-day or 11-day regimen) or doses (100 or 200 mg), showed a substantial 50–60% restoration in lung catalase activity (*p* < 0.05). In contrast, dexamethasone treatment failed to prevent the decrease in lung catalase activity induced by CSE (*p* = 0.7113)

### 3.4. Effect of NADH on CSE-Induced Changes in Inflammatory Cytokines

**Figure 5:**
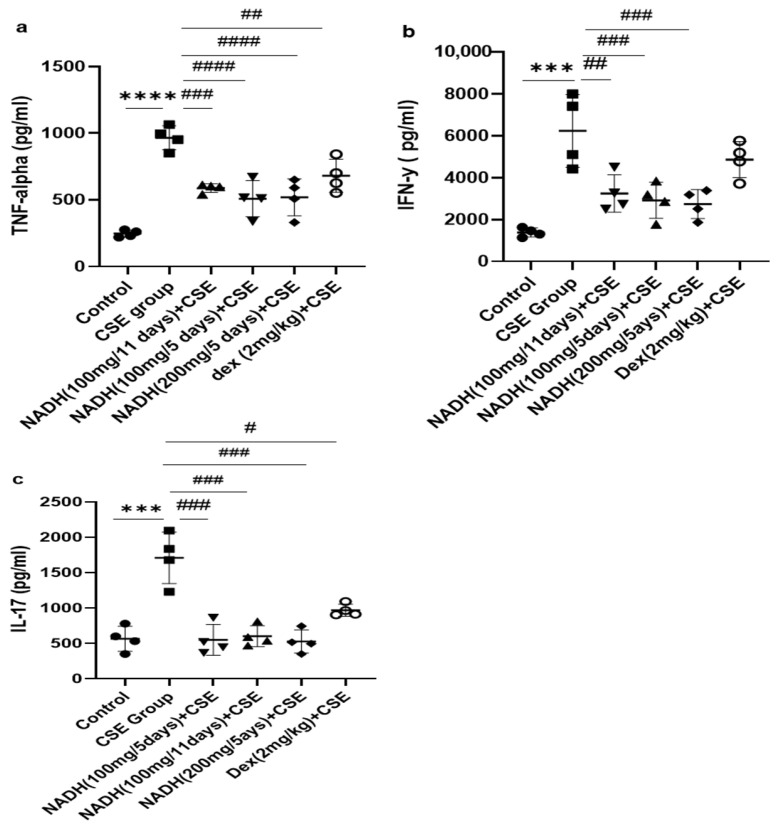
The effects of NADH on levels of TNF-alpha, IFN-γ, and IL-17 in the lungs of mice exposed to CSE. The levels of cytokines, including TNF-alpha (**a**), IFN-γ (**b**), and IL-17 (**c**), were assessed in lung homogenates from the control group or the groups treated with CSE alone or with NADH in the 5-day or 11-day regimen as described in the Section 2. *** *p* < 0.001 and **** *p* < 0.0001 compared with the control group; # *p* < 0.05 ## *p* < 0.01, ### *p* < 0.001, and #### *p* < 0.0001 compared with the CSE group (*n* = 4/per group).

As demonstrated in Figure 5a, we found that CSE induced a significant increase in the TNF-alpha level in the lung homogenates (3.9-fold) when compared with the control group (*p* < 0.0001). Treatment with 100 mg of NADH significantly reduced CSE-induced increases in TNF-alpha in all treated groups. The reduction was more pronounced among the groups that were treated every 5 days with 100 mg and 200 mg of NADH, respectively (47% vs. 40% inhibition; *n* = 4/per group). Additionally, NADH showed a better anti-inflammatory effect when compared to the positive control drug dexamethasone, which inhibited CSE-induced TNF-alpha only by 30% (*p* = 0.0004).

We also investigated the impact of NADH on CSE-induced IFN-γ production. The model led to a 4.5-fold increase in the IFN-γ level when compared to the control group (*p* < 0.001), as shown in Figure 5. All NADH-treated groups exhibited a marked decrease of 40–50% in the levels of IFN-γ (*p* < 0.001), while no effect was seen in the dexamethasone-treated group (*p* = 0.2874) (Figure 5b).

As depicted in Figure 5, CSE led to a three-fold increase in the IL-17 levels in the lungs when compared with the control groups (*p* < 0.0001). All groups treated with NADH, regardless of the treatment regimen (5-day or 11-day regimen) or dosage (100 or 200 mg), revealed a significant ~70% diminution of IL-17 (*p* < 0.001, *n* = 4). Conversely, dexamethasone only reduced by 40% the increased levels of lungIL-17 induced by CSE (*p* = 0.0194, *n* = 4) (Figure 5c).

### 3.5. Effect of NADH on CSE-Induced Inflammatory Changes in Lungs

**Figure 6:**
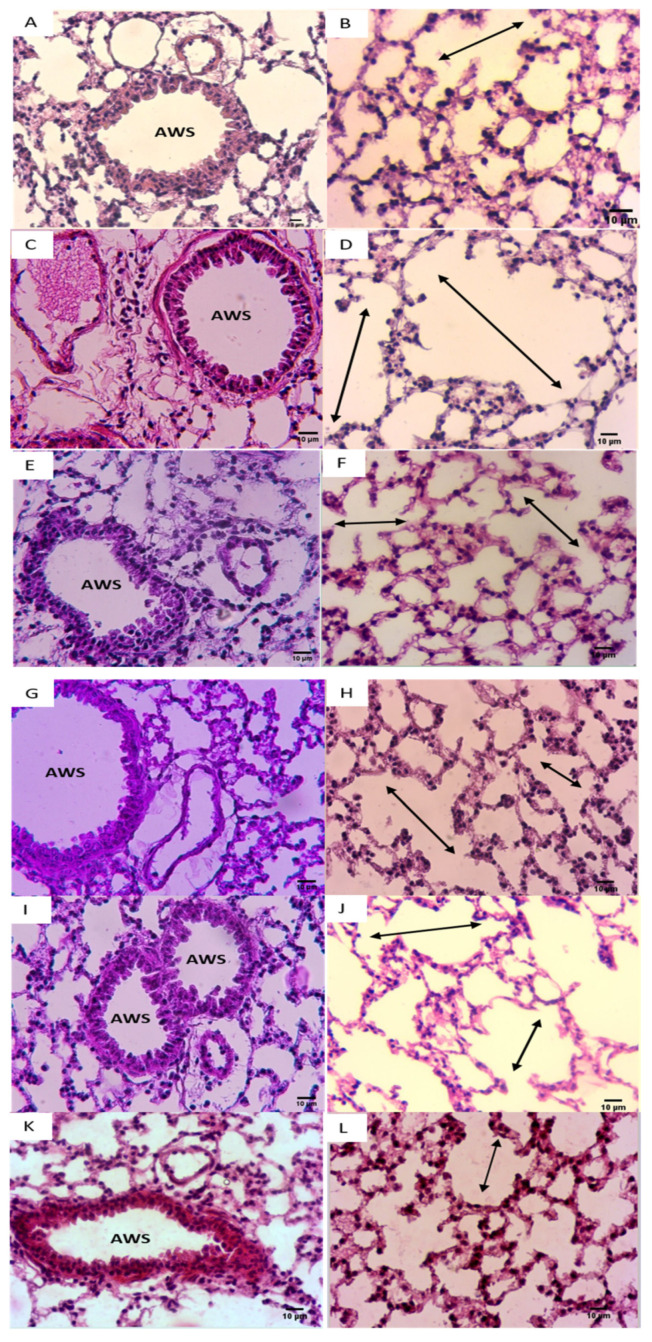
NADH ameliorated CSE-induced air space enlargement. Representative lung tissue sections (airways and parenchyma) stained with hematoxylin–eosin (H&E), taken at 400× magnification. (**A**,**B**) Control group; (**C**,**D**) CSE group. (**E**,**F**) NADH (100 mg/11 days) + CSE group. (**G**,**H**) NADH (100 mg/5 days) + CSE group. (**I**,**J**) NADH (200 mg/5 days) + CSE group. (**K**,**L**) Dexamethasone (2 mg/kg) + CSE group. Images are representative of *n* = 4 animals per condition. AWS = airways; double headed arrow = alveolar space enlargement.

CSE exposure was also associated with typical emphysematous changes in the lungs, characterized by an enlarged alveolar space with a very thin alveolar septum (Figure 6D) when compared with the control group (Figure 6B). In addition, CSE caused a well-defined arteriolar congestion with a moderate inflammatory infiltrate (Figure 6C). This inflammatory cluster was observed in the perivascular region with the well-defined alveolar changes mimicking emphysematous lesions (Figure 6C,D).

It is worth mentioning that these lung inflammatory changes coupled to emphysematous injuries were not observed in the NADH-treated groups (Figure 6C,E–J), further supporting the anti-inflammatory effect of NADH supplementation in this animal model of COPD.

**Figure 7:**
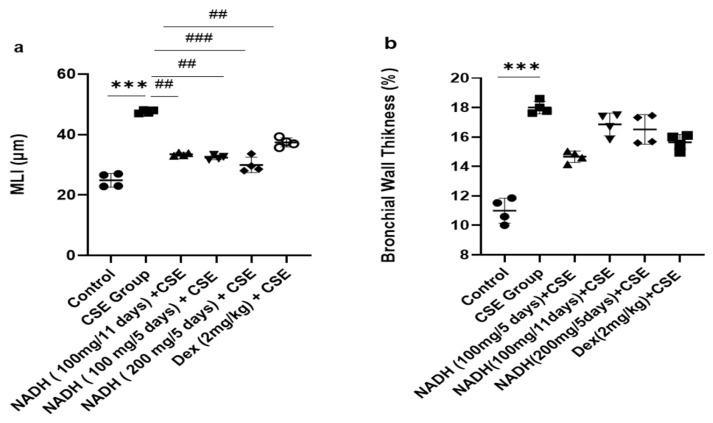
Lung morphometric analysis following CSE exposure in different treatment groups. Mean linear intercept (MLI) (**a**) and bronchial wall thickening (**b**) were calculated using Image J-v1.54j software. *** *p* < 0.001 versus control group, ## *p* < 0.01 and ### *p* < 0.001 vs. CSE group (*n* = 4/condition).

The 1.9-fold increase in MLI induced by CSE was equally reduced in the groups that were treated with 100 mg of NADH in the 5-day and 11-day regimens (*p* = 0.0014) and the group treated with 200 mg of NADH in the 5-day regimen (*p* = 0.0007, Figure 7).

Dexamethasone treatment induced a moderate protection from CSE-induced emphysema when compared with the control group with a 21% reduction in the MLI (*p* = 0.0098, Figure 7a). In addition, CSE also induced a marked increase in airway wall thickening (63% increase, *p* < 0.012, Figure 7b), which was not affected by NADH or dexamethasone treatment.

**Figure 8:**
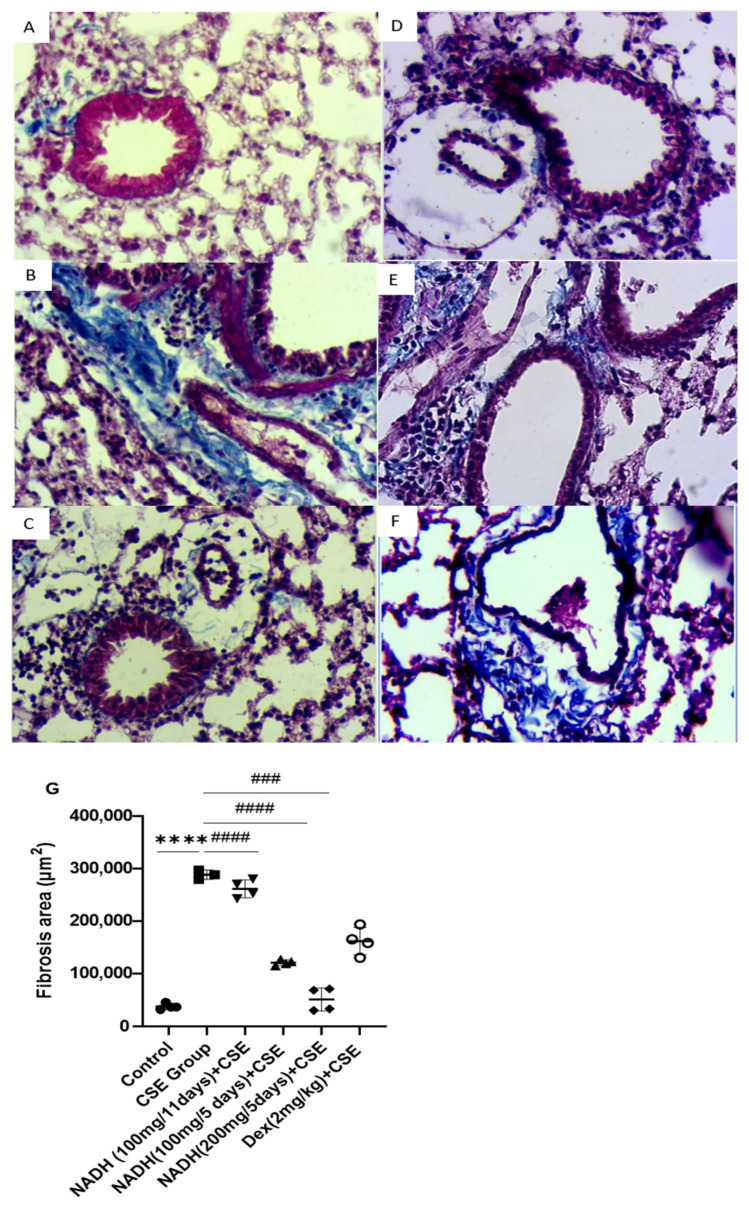
NADH protected against CSE-induced airway fibrosis. Representative lung tissue sections stained with Masson’s trichrome, taken at 400× magnification. Top panel: (**A**) control group, (**B**) CSE group, (**C**) NADH (200 mg/5 days) + CSE, (**D**) NADH (100 mg/5-day regimen) + CSE, (**E**) NADH (100 mg/11-day regimen) + CSE, (**F**) dexamethasone (2 mg/kg) + CSE, (**G**) assessment of collagen content. Bottom panel: comparison of extent of airway fibrosis (μm^2^) in lungs of different experimental groups compared to control group. **** *p* < 0.05 versus control group, ### *p* < 0.01 and #### *p* < 0.0001 versus CSE group, *n* = 4/per group.

NADH was also found to protect against CSE-induced lung fibrosis in the 5-day treatment regimen at both doses of 100 mg and 200 mg (*p* < 0.0001), leading to respective 58% and 82.5% decreases in lung fibrosis. In contrast, the 11-day regimen at a 100 mg dose did not provide any protection (*p* = 0.6888, Figure 8B), while dexamethasone protected against CSE-induced fibrosis by 43% compared with the CSE group, with *p* = 0.0006 (Figure 8B).

## 4. Discussion

There is no cure for patients with COPD, and the current available therapies (i.e., bronchodilators and steroids) do not treat the root of the problem nor do they reduce disease progression [31]. There is therefore an unmet need for novel therapies. We provide the first evidence that NADH therapy is effective in preventing the development of key pathological features associated with COPD, including airway emphysema, markers of oxidative stress, inflammation, and pulmonary fibrosis.

With cigarette smoke being the greatest risk factor for the development of COPD and emphysema, several models to mimic the disease have been developed by exposing animals to cigarette smoke (CS) either via the nose or via whole-body exposure [32,33]. Initial studies with such models have relied on various exposure protocols based on smoking apparatus involving different administration regimens, numbers of cigarettes, exposure routes (nose-only or whole-body exposure), and treatment duration (from weeks to several months) [34,35]. For example, notable differences were seen between nose-only and whole-body exposure in terms of the degree of lung inflammation and airway remodeling when side-by-side comparisons were made [36]. This suggests that the choice of animal models of COPD should really depend on the research question being asked. Additional models relying on intraperitoneal injection (IP) of cigarette smoke extracts (CSEs) have been also developed, with animals not only exhibiting lung manifestations of COPD such as inflammation, emphysema, and mucus hypersecretion, but also the systemic features of the disease such as heart and skeletal muscle dysfunction [37]. The IP injection model can also be established without the requirement of a longer treatment time (usually 3–6 weeks) [38,39]. Recent studies comparing CS exposure with IP injection models demonstrated comparable preclinical outcomes when looking at key COPD features, including the extent of lung damages (emphysema) and lung inflammatory changes [19,40,41,42]. Nonetheless, mice exposed to CSE represent one of the most commonly used models of COPD [34,35,39]. Here, our CSE-injection-based model of COPD given over the course of two months was able to generate clinical features of COPD, including emphysematous abnormalities, airway inflammation, and bronchial remodeling with marked lung fibrosis, as previously reported [43,44]. We relied on a combination of different parameters, such as WBC counting, oxidative stress evaluation, as well as lung morphological examination to confirm the validity of this inducible model of COPD. The choice of the treatment regimen (CSE injected every 11th day) was mostly based on previous reports showing the success of this exposure protocol to create COPD pathological features in the lung, including emphysema (94% of lung destruction) and lung inflammation [19,38,45].

**Figure 9:**
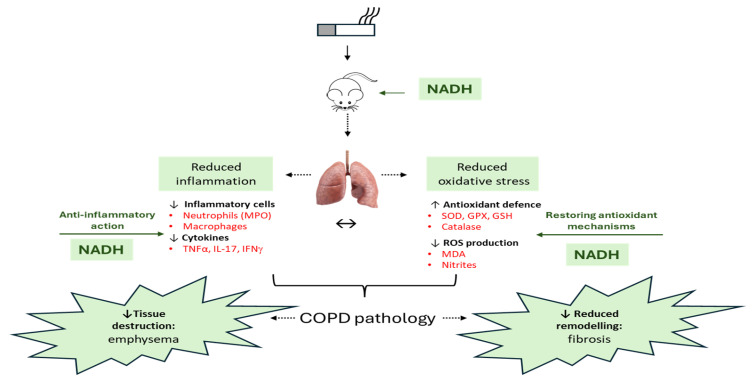
An overview of the therapeutic effect of NADH in the murine COPD model induced by systemic CSE exposure. The NADH-treated groups presented with a significant reduction in COPD key features, such as emphysema and airway remodeling (fibrosis). The therapeutic benefit of NADH is believed to result from blunted inflammatory pathways, as evidenced by the marked reductions in systemic granulocytes and lung neutrophils (reflected by decreased MPO levels). A significant decrease in the key COPD inflammatory cytokines (TNF-a, IFN-g, and IL-17) in the lungs may, in part, explain NADH’s beneficial action. These cytokines and other growth factors (TGF-b and CTGF) are known to play central roles in the chemoattraction and activation of macrophages/monocytes and neutrophils, thereby contributing to the amplification/perpetuation of inflammation/oxidative stress/remodeling processes in lung tissues. NADH supplementation was also found to markedly reduce CSE-induced oxidative damage, as shown by the diminution of typical biomarkers (MDA and nitrites) and the prevention of CSE-associated depletion of multiple antioxidant defense mechanisms (GPX, SOD, GSH, and catalase). ↔: Indicates the impact of NADH on both airway inflammation and oxidative stress may explain its beneficial action in COPD. GPX: glutathione peroxidase, GSH: glutathione, MDA: malondialdehyde, MPO: myeloperoxidase, SOD: superoxide dismutase. The image of the lung is from Pixabay.

Our overall finding was to investigate the potential clinical value of NADH supplementation as a preventive option in our murine model of COPD (Figure 9). This study was mostly fueled by the emerging evidence showing that an imbalanced NAD+/NADH ratio led to cellular homeostasis destabilization, which could be a driving factor for a number of chronic diseases such as diabetes [12], neurodegenerative diseases [13], and idiopathic lung fibrosis [14]. To our knowledge, no study has yet investigated the effect of NADH supplementation in any lung disease models. Here, we found that NADH supplementation at two different doses (100 and 200 mg given 30 min prior to CSE) almost completely prevented the key characteristics of COPD, including airway fibrosis (>82.5% reduction, Figure 8) as well as the structural features of emphysematous damages (>37% reduction, Figure 7) and inflammation (*p* < 0.001) that were seen in the lungs of CSE-exposed animals. Our study is in line with growing pre-clinical reports searching for novel compounds capable of reversing these CSE-induced COPD lung features. Some compounds have recently been uncovered to successfully prevent CSE-induced lung damage, including astaxanthin (AXT), a keto-carotenoid derived from the oxidation of β-carotene [46]; YPL-001, a drug candidate mixture isolated from *P. rotundum* var. Subintegrum [47]; aurintricarboxylic acid (ATA), a polyanionic aromatic compound [48]; and Retinoid X receptor agonists (RXRs) [49], all via multiple mechanisms. We believe that the protective action of NADH in our CSE model is derived from its ability to prevent mitochondrial dysfunction, which is often associated with high ROS production and oxidative stress. Indeed, we found that NADH at 100 mg and 200 mg doses in the 5-day regimen was highly effective in preventing the development of oxidative stress in the lungs by CSE, as evidenced by the marked drop in the levels of MDA and nitrites. In addition, NADH also completely prevented the loss of protective antioxidant mechanisms induced by CSE with the restoration of basal activities of SOD, GPX, GSH, and catalase.

Our current data show that NADH exerts strong antioxidant activities. The restoration of these defense barriers by NADH is believed to explain its beneficial effects in our CSE model of COPD. This hypothesis is supported by the marked reduction in key biomarkers of oxidative stress in the lungs of NADH-treated CSE-exposed animals, such as MDA, the end-product of lipid peroxidation. The mechanisms by which NADH supplementation prevented the loss of antioxidant pathways have not clearly been defined but could be related to the mono-ADP-ribosyltransferases, sirtuins, and PPAR-γ co-activator1α (SIRT/PGC-1α) pathways. Indeed, SIRT1 has been shown to exert several beneficial effects, including promoting mitochondrial biogenesis, preventing cell apoptosis, and restoring metabolic balance [50]. Interestingly, dexamethasone, used as our anti-inflammatory control drug, failed to reverse the loss of catalase and GPX, showing the superior clinical potential of NADH in our model (Figure 4).

We believe that NADH supplementation may impact inflammation and associated lung injury by preventing CSE-induced mitochondrial dysfunction reported as an important source of ROS and proinflammatory factors in COPD, such as TNFα/IL-1β, which led to the recruitment of neutrophils [51]. Several lines of evidence from our report support this assumption. First, NADH blunted the expression of oxidative stress markers and markedly reduced the increased levels of TNF-α, IFN-γ, and IL-17 in the lungs of CSE-exposed mice. TNF-α and IFN-γ, which are predominantly produced by activated macrophages and lymphocytes, can trigger the release of CXCL10, a chemokine attracting monocytes, neutrophils, and T-cells, notably Th1 cells, thereby contributing to pulmonary emphysema [48]. In addition, all of these cytokines are involved in COPD pathogenesis via their contributions to both lung structural abnormalities and the recruitment/activation of inflammatory cells [49]. For example, a recent systemic review and meta-analysis study conducted with *n* = 2268 patients confirmed a critical role of IL-17 in driving neutrophilic inflammation and exacerbation in COPD [52]. It is not surprising to see that reducing lung levels of IL-17 by NADH was associated with a parallel decrease in lung MPO activity, an heme-containing peroxidase mainly expressed neutrophils [53]. Although we did not measure the neutrophil count in the lungs, we saw a complete drop in the total number of granulocytes recruited by CSE in the blood via NADH treatment. This again suggests that NADH therapy is a strong modulator of neutrophilic inflammation in lungs exposed to cigarette smoke. Other investigations encompassing both animal and clinical studies have emphasized the role of IL-17, primarily originating from CD4+ Th17 cells, in regulating the recruitment of macrophages and neutrophils, as well as modulating chemokine activity. Moreover, IL-17 has been closely associated with the development and progression of COPD [3,52].

Our study supports the conclusion from previous reports suggesting the implication of mitochondrial dysregulation in the pathogenesis of COPD/emphysema [54] and a pivotal role of cigarette smoke in inducing oxidative damage in the lungs as a result of perturbating mitochondrial function [8]. Manipulating NADH levels by pharmacological agents has the potential to restore mitochondrial respiration and showed promising results in the treatment of diabetes and multiple disorders [15], chronic fatigue syndrome [16], and neurodegenerative diseases [17,18]. NAD treatment reduced the activation of circulating immune cells through the blockage of NLRP/3 inflammasome formation and inflammatory cytokine production. Furthermore, NAD supplementation leads to the activation of sirtuins 1 and 2, which inhibit the transcription factor NF-κB [55,56] resulting in a decrease in the gene expression of pro-inflammatory cytokines [57]. As the reduced form of NAD, NADH can act as the major source of its intracellular regeneration [58,59]. The relationship between lung fibrosis and oxidative stress in COPD has not been clearly established. The profibrotic responses occurring in the lungs of CSE-treated animals may result from the abnormal production/activation of growth factors, such as TGFβ/CTGF, which are reported to be increased in the lungs of patients with COPD [60]. Indeed, in addition to promoting oxidative stress and the proliferation of lung fibroblasts/myofibroblasts [61], TGFβ has been reported to interfere with the activity of Nrf2, a central transcription factor in the induction of endogenous antioxidant proteins, further enhancing oxidative stress [62]. Our model of experimental COPD showed that lung fibrosis was closely associated with reduced levels of many Nrf2-inducible antioxidant proteins (i.e., SOD, catalase, and GSH) in the lungs of CSE-treated animals, and whether this was due to the action of growth factors remains an interesting hypothesis to be examined in future studies. However, lung fibrosis in COPD is believed to be a complex mechanism resulting from the chronic inflammatory environment as well as self-perpetuating oxidative microenvironment due to an oxidative stress imbalance [63].

The concept that either NAD+ or NADH cannot cross the plasma membrane has to be revisited based on some evidence demonstrating that extracellular NADH can be transported across the plasma membrane via a P2X7R-dependent mechanism in astrocytes to generate NAD+. The authors used the P2X7 receptor blocking strategy (antagonism and knockdown) to demonstrate that the receptor was essential in the NADH-induced accumulation of NAD+ inside the cells [64]. Interestingly, P2X7R is known to be expressed in several cells involved in lung diseases; it is not surprising to see that P2X7R antagonists have been suggested for the treatment of various respiratory conditions associated with lung inflammation and fibrosis [65]. In fact, the P2X7 receptor was shown to be essential in cigarette smoke-induced lung inflammation and emphysema in mice [66]. Because the P2X7 receptor was increased by cigarette smoke exposure in key inflammatory cells such as airway neutrophils, alveolar macrophages, and in whole lung tissues, it is tempting to speculate that this would be associated with an increased uptake of NADH levels in the lungs, thus potentially explaining its local therapeutic effects. Additional studies are needed to confirm this interesting hypothesis. Similarly, extracellular NAD+ was also reported to induce different cellular effects, such as nucleotide metabolism and genomic DNA replication, when exposed to cultured cell models [67]. Similar to NADH, NAD+ has been shown to be transported across the plasma membrane via different transporter proteins, with one being connexin 43 hemichannels [68,69]. These studies strongly suggest that both extracellular NAD+ and NADH have the capacity to cross the plasma membrane via mechanisms that remain to be further explored.

Our present study opens a completely new area of investigation, namely the study of the supplementation of NADH and NAD+ precursors, including nicotinamide (NAM), nicotinamide riboside (NR), and nicotinamide mononucleotide (NMN), as potential therapies in the treatment and/or prevention of COPD features. It would also be important to investigate the cellular and molecular mechanisms underlying the protective effect of NADH by looking at the factors mediating its antioxidant and anti-fibrotic actions. To further support the hypothesis that COPD is indeed associated with the dysregulation of NAD+ pathways, it would be important to determine whether the levels of NAD+ are reduced in patients with COPD and/or whether reduced levels correlate with disease severity or the rate of exacerbations. It would also be essential to determine the impact of NADH supplementation when given therapeutically, i.e., once the disease is already established, rather than preventively, as given in the present study. Lastly, testing a strategy that combines NADH/NAD precursors and current therapies (LABA, long-acting β2-agonists, or steroids, for example) in preclinical models of COPD may reveal a superior therapeutic effect that can be exploited in clinics. This is a clinically relevant area worth pursuing as different cohort studies have reported perturbed NAD pathways in aging and age-related conditions coupled to the strong preclinical evidence showing the effectiveness of NAD+ precursor supplementation in numerous animal models of human conditions. In addition, there are various ongoing clinical trials testing the supplementation of NAD+ precursors in various healthy and disease states [57]. Our studies support the use of the NAD+/NADH replenishing strategy in the management of patients with COPD.

## 5. Conclusions

Our study provides the first evidence of a protective role of NADH in preventing the development of key pathological features associated with COPD, including airway emphysema, markers of oxidative stress, inflammation, and pulmonary fibrosis.

## Data Availability

The data presented in this study will be made available upon request from the corresponding author.
